# Unexpected Dominance of Elusive Acidobacteria in Early Industrial Soft Coal Slags

**DOI:** 10.3389/fmicb.2017.01023

**Published:** 2017-06-08

**Authors:** Carl-Eric Wegner, Werner Liesack

**Affiliations:** ^1^Department of Biogeochemistry, Max Planck Institute for Terrestrial MicrobiologyMarburg, Germany; ^2^Aquatic Geomicrobiology, Institute of Ecology, Friedrich Schiller University JenaJena, Germany

**Keywords:** soft coal, slags, mineral leaching, acid mine drainage, microbiome, acidobacteria, metagenomics, microbial dark matter

## Abstract

Acid mine drainage (AMD) and mine tailing environments are well-characterized ecosystems known to be dominated by organisms involved in iron- and sulfur-cycling. Here we examined the microbiology of industrial soft coal slags that originate from alum leaching, an ecosystem distantly related to AMD environments. Our study involved geochemical analyses, bacterial community profiling, and shotgun metagenomics. The slags still contained high amounts of alum constituents (aluminum, sulfur), which mediated direct and indirect effects on bacterial community structure. Bacterial groups typically found in AMD systems and mine tailings were not present. Instead, the soft coal slags were dominated by uncharacterized groups of Acidobacteria (DA052 [subdivision 2], KF-JG30-18 [subdivision 13]), Actinobacteria (TM214), Alphaproteobacteria (DA111), and Chloroflexi (JG37-AG-4), which have previously been detected primarily in peatlands and uranium waste piles. Shotgun metagenomics allowed us to reconstruct 13 high-quality Acidobacteria draft genomes, of which two genomes could be directly linked to dominating groups (DA052, KF-JG30-18) by recovered 16S rRNA gene sequences. Comparative genomics revealed broad carbon utilization capabilities for these two groups of elusive Acidobacteria, including polysaccharide breakdown (cellulose, xylan) and the competence to metabolize C1 compounds (ribulose monophosphate pathway) and lignin derivatives (dye-decolorizing peroxidases). Equipped with a broad range of efflux systems for metal cations and xenobiotics, DA052 and KF-JG30-18 may have a competitive advantage over other bacterial groups in this unique habitat.

## 1. Introduction

Mining frequently produces enormous amounts of tailings, which often pose the danger of generating acid mine drainage (AMD) (Rohwerder et al., 2003). AMD is a consequence of sulfur- and iron-rich minerals, primarily pyrite (FeS_2_), being exposed to oxygen and humidity (Druschel et al., 1999). Pyrite oxidation leads to the formation of sulfuric acid and ferrous iron. Sulfuric acid lowers the pH and ferrous iron is further oxidized yielding ferric iron. The gradually decreasing pH increases the solubility of ferric iron, which is a stronger oxidant of pyrite than oxygen (Druschel et al., 1999). Microbial activity accelerates AMD generation tremendously. Microbial iron and sulfur oxidation are orders of magnitude faster than the abiotic oxidation of these two elements. The microbial activity easily leads to a release of acidic and metal-contaminated discharges. Early studies targeting AMD microbial communities revealed the prevalence of a few taxonomic groups, including *Leptospirillum* and *Acidithiobacillus* among the bacteria and *Ferroplasma* and other *Thermoplasma*-related groups (A-, E-, and G-Plasma) within the archaea (Baker and Banfield, 2003). Metagenomic (Tyson et al., 2004; Dick et al., 2009; Yelton et al., 2011, 2013), metatranscriptomic (Lehembre et al., 2013; Hua et al., 2015; Chen et al., 2015) and metaproteomic (Denef et al., 2010; Mueller et al., 2011) studies showed that iron- and sulfur-cycling are the dominating microbial activities in AMD environments. Most AMD studies focused either on tailings as source material (Radeva and Selenska-Pobell, 2004; Senko et al., 2008; Urbanová et al., 2011; Korehi et al., 2014) or on AMD discharges as the end product (Baker and Banfield, 2003; Tyson et al., 2004; Dick et al., 2009; Xie et al., 2011; Kuang et al., 2013). The most common sources of AMD are mine tailings, and iron and coal deposits.

Though coal is nowadays almost exclusively mined for its use as fossil fuel, mineral-rich coal is mined to win scarce and precious minerals such as germanium (Arroyo and Fernández-Pereira, 2008). In the nineteenth century, sulfur mineral-rich soft coal was mined in Western Germany, in the surroundings of Bonn, to extract these minerals by a smelting-like process (Supplementary Figure 1). Among these minerals, especially alum (KAl(SO_4_)_2_×12H_2_O) was commonly used as mordant and precipitant in early textile and paper industry. Alum was extracted from soft coal in a four-step process. Soft coal was mined, sheared and subsequently smoldered to enrich present alum. The resulting ash (one third of the original coal, alum content 15%) was leached and the leachate boiled to yield crystalline alum. Overall, alum leaching was a low-yield process. One ton of coal had to be processed to win 50 kg of crystalline alum, thereby leading to 300–400 kg of leached slag as by-product. At peak year level, 500,000 tons of coal were mined, producing 1,250 tons of alum and 150,000–200,000 tons of slag. In the middle of the nineteenth century, the alum extraction plants and soft coal mines in the surroundings of Bonn were the biggest of their kind in former Prussia. Given a mining period of roughly 70 years (1805–1875), tremendous amounts of leached slag were produced and dumped into the environment without precautions and remediation.

Here we investigated the microbiology of soft coal slag deposits. Given their industrial history and leached nature, we hypothesized that the slags represent an endpoint AMD environment that is characterized by a unique bacterial community composition. In particular, we were interested in exploring to which extent the remaining alum and other metals affect the genome coding potential of the slag-deposit-inhabiting microbial communities. An additional challenge faced by these communities is the recalcitrant nature of their carbon sources. Our research involved a thorough geochemical analysis, bacterial community profiling, and shotgun metagenomics. In addition to samples from three slag deposit sites, sediment from a drainage pond and nearby undisturbed forest soil were examined for comparison.

## 2. Materials and methods

### 2.1. Sampling

Detailed information about the sampling site, sampling and alum leaching from soft coal can be found in the Supplementary Methods and Supplementary Figure 1. Three individual sites were sampled at the slag deposit “Red Hill” (abbreviated: RH1 [50° 44′21.45″N, 7° 10′27.87″E], RH2 [50° 44′21.85″N, 7°10′27.78″E], RH3 [50° 44′22.23″N, 7° 10′27.46″E]) in September 2014. After removing a cover layer (approx. 5 cm) of slowly humifying biomass, the underlying slag was collected in 1 L plastic containers. Two additional sites were sampled for reference and comparison: Sediment of a nearby pond, which collects the drainage water of slag deposits upon rain events (abbreviated: RHP [50° 44′22.32″N, 7° 10′28.32″E]) and nearby undisturbed forest soil (abbreviated: Ref [50° 43′49.63″N, 7° 10′30.44″E). Three replicate samples were collected within a 4 m^2^ area surrounding each individual sampling site. Replicates were pooled to obtain composite samples that were aliquoted and subjected to individual analyses. Geochemical analyses were carried out as described in the Supplementary Methods.

### 2.2. DNA extraction

DNA was extracted using the FastDNA® SPIN kit for soil (MP Biomedicals, Eschwege, Germany). Quality and integrity of the DNA was assessed by agarose gel electrophoresis. DNA concentrations were determined fluorometrically. Extracts were aliquoted and stored at −20° C for downstream analyses, including qPCR, amplicon sequencing and shotgun metagenomics.

### 2.3. 16S rRNA gene-targeted qPCR

Bacterial 16S rRNA gene copies were determined by SybrGreen-based quantitative PCR (Stubner, 2004). Standard curves were generated using genomic DNA from *E. coli* (calibration range: 10 – 10^6^ copies). Quantitative PCR was carried out using a CFX Connect Real-Time PCR detection system (Bio-Rad, Munich, Germany). The PCR efficiency was at least 85% (*R*^2^ ≥ 0.99). Melt curve analyses were carried out to check for unspecific products.

### 2.4. Amplicon library preparation and sequencing

PCR amplicons for subsequent amplicon library preparation were generated using a bacteria-specific (341f [S-D-Bact-0341-b-S-17, 5′-CCTACGGGNGGCWGCAG-3′]/805r [S-D-Bact-0785-a-A-21, 5′-GACTACHVGGGTATCTAATCC-3′]) primer pair (Klindworth et al., 2013). The PCR assay is further described in the Supplementary Methods. PCR products were checked by agarose gel electrophoresis, gel-excised and purified using the Wizard® SV Gel and PCR clean-up system (Promega, Mannheim, Germany). Library preparation was done using the NEBNext® Ultra DNA Library Prep Kit for Illumina® (New England BioLabs, Frankfurt/Main, Germany), with a final library size of 550 bp. The size distribution was checked by running DNA 12K chips on a Bio-Rad Experion® (Bio-Rad, Munich, Germany). Sequencing was carried out at the Max Planck Genome Centre Cologne using an Illumina® MiSeq platform in paired-end mode (2 × 300 bp).

### 2.5. Shotgun metagenome library preparation and sequencing

Extracts of total DNA were sheared to a fragment size of approximately 200–250 bp using a Bioruptor® Plus (Diagenode, Denville, New Jersey, USA). Shearing involved 15 cycles of 30 s/30 s (on/off) at high intensity. Library preparation was done as described above. Size selection was modified to yield a final library size of approximately 250–300 bp. Libraries were sequenced using an Illumina® HiSeq 2500 device in paired-end mode (2 × 100 bp) at the Max Planck Genome Centre Cologne.

### 2.6. Amplicon data processing

Amplicon data were quality-controlled and pre-processed as described in the Supplementary Methods. OTUs (operational taxonomic units) were clustered using USEARCH (v. 7.0) (Edgar, 2010) and a sequence identity threshold of 97%. A compatible OTU table was assembled to facilitate amplicon data analysis using QIIME (v. 1.8) (Caporaso et al., 2010) and PHYLOSEQ (v. 1.10) (McMurdie and Holmes, 2013).

### 2.7. Metagenome data processing and computational analysis

Quality control of metagenome reads was done as described for amplicon datasets (Supplementary Methods), except that initial paired-end assembly was omitted, and reads were not filtered based on expected errors but truncated from the first position showing a quality score below Q20. The taxonomic affiliation of quality-controlled reads prior to metagenome assembly was determined using KRAKEN (v. 0.10.5) (Wood and Salzberg, 2014) and UBLAST (an optimized BLAST (Altschul et al., 1997) algorithm implemented in USEARCH) searches against NCBI NR (*e*-value 1e^−3^) (Pruitt et al., 2012). Full-length 16S rRNA gene sequences were reconstructed using EMIRGE (Miller et al., 2011) and matched against the SILVA database (v. 123) (Quast et al., 2013) using ARB (v. 6.0.2) (Ludwig et al., 2004). The nearly complete 16S rRNA gene sequences allowed us to validate sufficient sequencing depth by detecting abundant groups previously identified by community profiling (Supplementary Figures 2, 3). Metagenome assembly was done with MEGAHIT (v. 1.0.1) (Li et al., 2014). After examining a variety of different k-mer lengths, a minimum k-mer length of 33 and a maximum k-mer length of 73 were applied with 10-mer iterations. Contigs greater than 1000 bp were taxonomically binned using PHYLOPYTHIA S+ (Gregor et al., 2016). This involved the analysis of marker genes and k-mer profiles. The coverage of individual contigs was calculated using BBMAP (v. 35.02) (http://sourceforge.net/projects/bbmap/). Coding genes were predicted using PRODIGAL (v. 2.6.2) (Hyatt et al., 2012).

### 2.8. Recovery of metagenome-assembled genomes

A two-step binning strategy was applied for the recovery of metagenome-assembled genomes (MAGs) (Supplementary Figure 4). The procedure is described in detail in the Supplementary Methods. MAGs were examined for completeness and possible sequence contamination by CHECKM (v. 1.0.3) (Parks et al., 2015). MAGs more complete than 85% were considered high-quality draft genomes, while those more complete than 70% were considered good-quality draft genomes. The categorization was based on the presence of lineage-specific single-copy marker genes and a sequence contamination of less than 10%. High- and good-quality draft genomes were subjected to genome annotation. MAGs of lesser quality were processed to determine their taxonomic affiliation. Genome annotations were done using PROKKA (v. 1.11) (Seemann, 2014) and RAST (v. 2.0) (Aziz et al., 2008; Overbeek et al., 2014) and validated against each other. Annotated genomes were analyzed using ARTEMIS (v. 16.0) (Carver et al., 2012). Gene predictions were examined by parsing the output from UBLAST searches in MEGAN (v. 5) (Huson et al., 2011) and by generating KEGG (Kyoto Encyclopedia of Genomes and Genes) annotations by subjecting coding genes to GHOSTKOALA (v. 2.0) (Kanehisa et al., 2015). The phylogenetic affiliation of recovered MAGs was determined by identified 16S rRNA genes and by concatenated alignments of 31 single-copy marker genes using AMPHORA (v. 2.0) (Wu and Scott, 2012). A maximum-likelihood tree was calculated using FASTTREE (v. 2.1.3) (Price et al., 2010) and the WAG model (Whelan and Goldman, 2001). Nearest neighbor interchange was applied for optimizing tree topology and the CAT approximation was used to account for evolutionary rate heterogeneity (Stamatakis, 2014). Genes of interest (e.g., genes encoding Czc efflux pumps) were extracted from the genomes (nucleotide sequences and deduced amino acid sequences) and queried against Interpro (v. 62.0), Pfam (v. 30.0) and Tigrfam (v. 15.0) for more detailed analyses (Haft et al., 2003; Mitchell et al., 2015; Finn et al., 2016).

### 2.9. Statistical analyses

Statistical analyses were carried out using the R software framework (v. 3.2.2) (R Development Core Team, 2008) and the packages APE (v. 3.3) (Paradis et al., 2004), METAGENOMESEQ (v. 1.13.02) (Paulson et al., 2016), PHYLOSEQ (v. 1.10) (McMurdie and Holmes, 2013), and VEGAN (v. 2.3.1) (Oksanen et al., 2016), including their respective dependencies. Individual analyses are described in more detail in the Supplementary Methods.

### 2.10. Figure generation

Figures were generated using the R packages GGPLOT2 (http://ggplot2.org) (Wickham, 2009), as well as the python module MATPLOTLIB (http://matplotlib.org/) (Hunter, 2007).

### 2.11. Sequence data deposition

Amplicon sequencing and shotgun metagenomics data, and genome bins of interest were deposited at NCBI SRA and NCBI Genomes and are accessible under the following BioProject/Genome accessions: PRJNA384238 (amplicon sequencing data), PRJNA384362 (shotgun metagenomics data), NFUN00000000 (RH2 MAG17b), NFUO00000000 (RH1 MAG20).

## 3. Results

### 3.1. Bacterial community profiling

Both 16S rRNA gene-targeted quantitative PCR and DNA extraction efficiency showed that the recovery of microbial biomass was 10-fold lower for slag samples RH1-3 (2.20−2.89 × 10^8^ copies/g dry weight [DW] soil; 4.45–8.24 μg DNA/g DW) than for the undisturbed forest soil (Ref: 2.21 × 10^9^ copies/g DW, 45.18 μg DNA/g DW). The corresponding values for RHP were as follows: 1.51 × 10^8^ copies/g DW, 22.53 μg DNA/g DW (Supplementary Figure 5). Chao1 and Shannon indices revealed that alpha diversity and evenness of the bacterial communities in slag deposits and pond sediment were lower than in undisturbed forest soil (Supplementary Figure 6). Lowest alpha diversity and evenness values were calculated for RH2 and RH3. Beta diversity analysis showed that the bacterial communities in samples RH1-3, RHP, and Ref differed in structural composition (Supplementary Figure 6). In particular, the communities in slag deposits (RH1-3) clustered clearly distinct from those in pond sediment (RHP) and forest soil (Ref), as revealed by Jensen-Shannon divergences.

**Figure 1:**
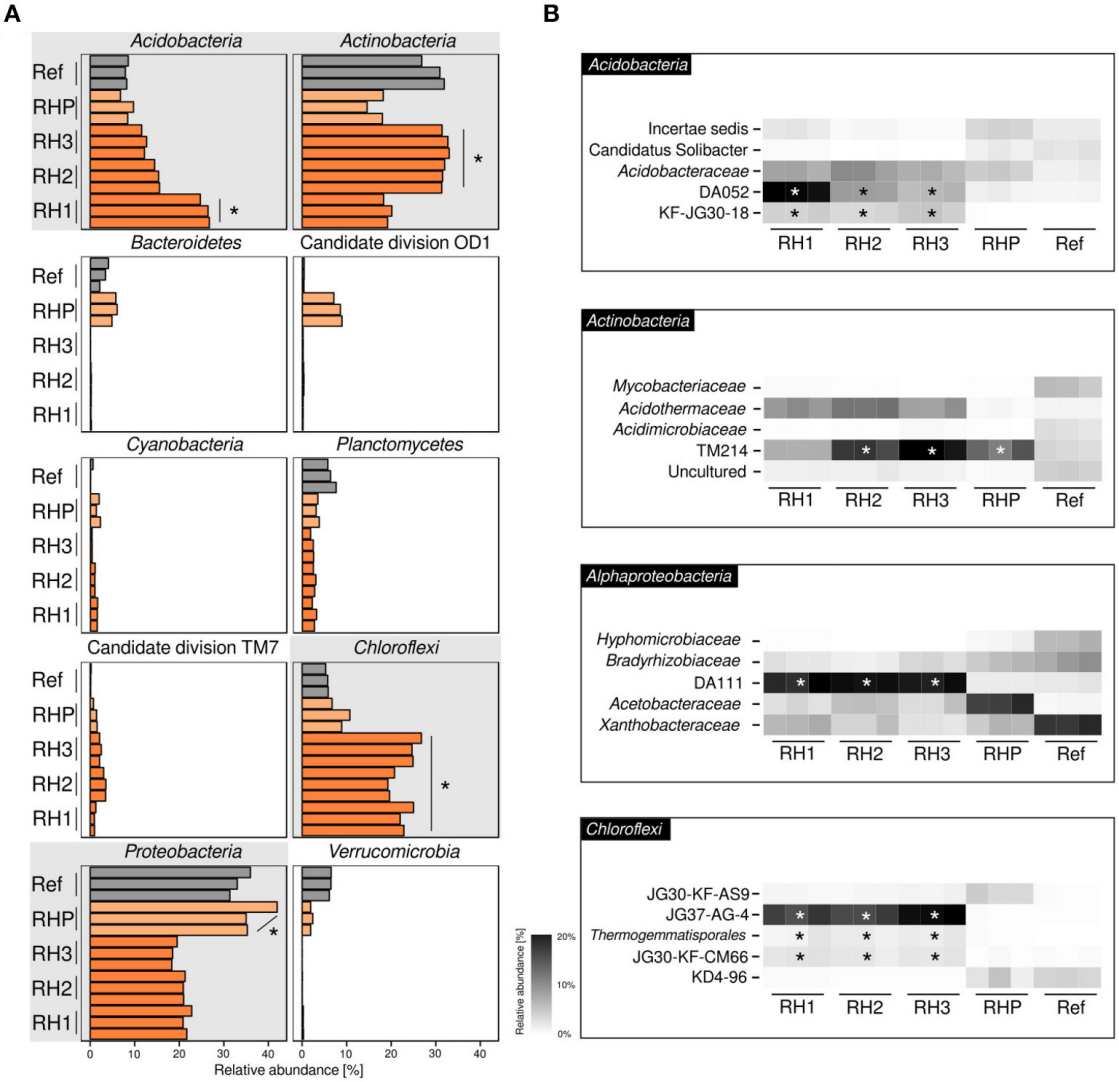
Bacterial community profiling based on amplicon sequencing of bacterial 16S rRNA genes. Community profiles were obtained for three slag deposit sites (RH1-RH3), sediment of a pond collecting the slag drainage water of rainfall events (RHP) and nearby undisturbed forest soil (Ref). **(A)** Phylum-level analysis is shown for the ten most abundant bacterial groups. Panels linked to the four phyla showing highest abundances are emphasized in gray. Orange bars = RH1-3, light-orange bars = RHP, gray bars = Ref. **(B)** Taxonomically more resolved community profiles for the four most abundant phyla. ^*^Significant enrichment (*p* ≤ 0.05) based on one-way ANOVA.

Basic characteristics of amplicon datasets are given in Supplementary Table 1. Bacterial communities were dominated by four phyla (range of averaged relative abundances in sample-specific amplicon sequencing datasets are given in parenthesis): Acidobacteria (6.8–26.8%), Actinobacteria (14.6–33.0%), Chloroflexi (5.3–26.8%) and Alphaproteobacteria (18.3–42.0%) (Figure 1A). Acidobacteria and Actinobacteria showed a divergent pattern, with Acidobacteria being enriched in site RH1 (ANOVA, *p* ≤ 0.05), while Actinobacteria were most prevalent in sites RH2 and RH3. Chloroflexi showed increased abundances in slag deposits (RH1-3, *p* ≤ 0.05), while Alphaproteobacteria were strongly enriched in pond sediment (RHP). Less-abundant phyla included Planctomycetes (1.8–7.6%), Cyanobacteria (0.1–1.6%), and Bacteroidetes (0.1–6.0%). The latter groups, however, were detected only in sites RHP and Ref. A more detailed taxonomic analysis showed that all four dominant phyla were represented mainly by uncharacterized order- and family-level groups (Figure 1B). These groups included: DA052, KF-JG30-18 (Acidobacteria [subdivision 2, subdivision 13]), TM214 (Actinobacteria [Acidimicrobiales]), DA111 (Alphaproteobacteria [Rhodospirillales]), and JG37-AG-4 (Chloroflexi). Both DA052 and KF-JG30-18 were significantly enriched in all three RH sites (*p* ≤ 0.05), with DA052 being most abundant in site RH1. TM214 was detected at all sites, but most pronounced at sites RH2, RH3, and RHP (*p* ≤ 0.05). Like DA052 and KF-JG30-18, DA111 and JG37-AG-4 were significantly enriched and showed high abundances in all slag samples.

In order to gain a more resolved phylogenetic analysis, we made the attempt to reconstruct full-length 16S rRNA gene sequences from our metagenomic datasets. We recovered nearly complete (≥ 1200 bp) 16S rRNA gene sequences for all the most abundant groups: DA052 (9 sequences), KF-JG30-18 (2), DA111 (10), JG37-AG-4 (13), and TM214 (22). Phylogenetic analysis showed that all five groups—DA052, KF-JG30-18, DA111, JG37-AG-4, and TM214—were related to 16S rRNA gene sequences previously recovered from acidic forest soil. DA052, KF-JG30-18 and TM214 also showed a close affiliation to uncultured bacteria detected in volcanically impacted soil systems and, in addition, TM214 to populations in iron mineral-rich environments. Uncultured relatives of DA052, DA111, and JG37-AG-4 were also detected in metal-contaminated sites (Supplementary Table 2).

### 3.2. Geochemical characteristics

**Figure 2:**
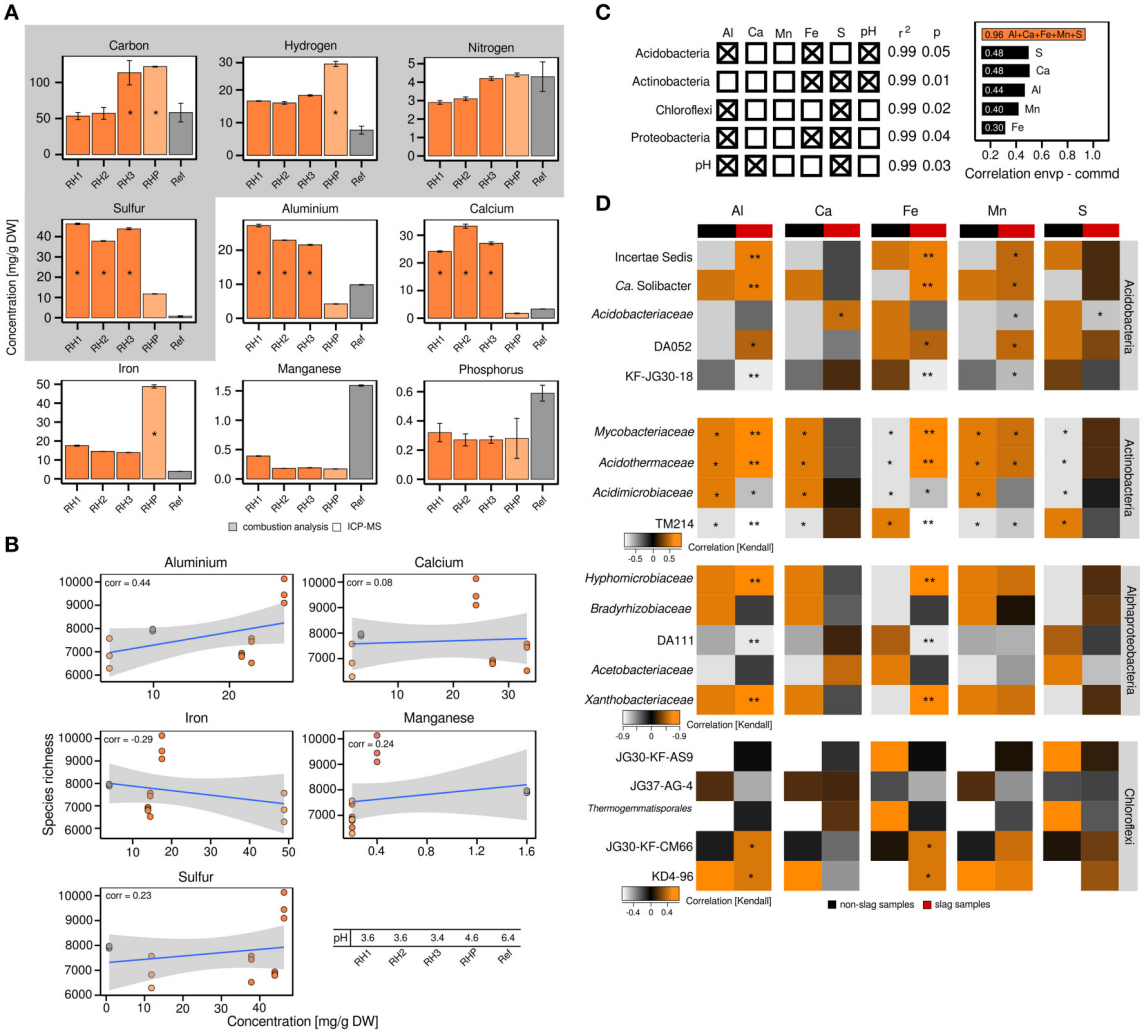
Correlation analyses between the abundance of particular bacterial taxa and elements. Detection and quantitation of the examined elements involved combustion analysis and inductively coupled plasma mass spectrometry, ^*^significant enrichment (*p* ≤ 0.05) based on one-way ANOVA, DW = dry weight **(A)**. Correlation analysis between bacterial species richness and particular elements based on Pearson correlation coefficients (corr = correlation) **(B)**. Multiple regression analysis based on log-transformed elemental data (left) and correlations between either individual environmental parameters or the complete set of all five environmental parameters and bacterial community structure as determined by relating the environmental parameters to determined Jensen-Shannon divergences (right; envp = environmental parameter, commd = community dissimilarity) **(C)**. Kendall's tau coefficients were calculated to link the occurrence of specific taxa within phyla of interest to selected environmental parameters. Determined *p*-values were corrected for multiple comparisons (Benjamini and Hochberg, 1995). Asteriks indicate ^*^p ≤ 0.05 and ^**^p ≤ 0.01 **(D)**.

The differences in bacterial community structure between RH1-3 and RHP/Ref prompted us to collect various metadata to place the community profiles in a broader context. Given the unusual industrial history of the slag deposits, we were especially interested in the metal content of the slag and the possible presence of xenobiotics. Elemental analysis revealed that the content of sulfur (37.33–46.31 mg/g DW soil), aluminum (21.57–27.23 mg/g), and calcium (24.12–33.26 mg/g) was significantly (ANOVA, *p* ≤ 0.05) increased in the slag deposits (RH1-3), relative to the undisturbed forest soil (Ref) (sulfur: 0.77 mg/g, aluminum: 9.85 mg/g, calcium: 3.32 mg/g) (Figure 2A). The pond site showed a strongly elevated level of iron (48.84 mg/g) (*p* ≤ 0.05) (RH1-3: 13.88–17.52 mg/g, Ref: 3.36 mg/g). BTEX compounds (benzene, toluene, ethylbenzene and xylene), PAHs (polycyclic aromatic hydrocarbons) and heavy metals were not detected. Samples from the slag deposits (RH1-3) had a low pH of 3.4–3.6. The pond sediment (RHP) was less acidic (Figure 2B).

The attempt to relate the observed alpha diversity at the individual sampling sites to particular environmental parameters (aluminum, calcium, iron, manganese, sulfur) showed no or poor correlations, with the exception of aluminum (correlation = 0.44) (Figure 2B). We, however, detected significant correlations between the abundance of particular bacterial phyla and environmental parameters, such as sulfur, iron and aluminum (*p* ≤ 0.05, *r*^2^ ≥ 0.99), based on multiple regression analyses (Figures 2C,D). The pH was not an independent parameter, but significantly linked to the available sulfur and aluminum (*p* ≤ 0.05, *r*^2^ ≥ 0.99). As revealed by Jensen-Shannon divergences, the correlations between the observed differences in bacterial community structure and environmental parameters were in the range of 0.3–0.48 (Figure 2C). Combining the different environmental parameters into a best-fit model, however, yielded a strong correlation of 0.96.

Given the dominance of yet-uncharacterized groups (Figure 1B) and greatest differences in both bacterial community profiles and environmental parameters between slag deposits (RH1-3) and non-slag samples (RHP + Ref), we determined Kendall's tau coefficients to identify links between particular taxonomic groups and single environmental parameters (Figure 2D). Among the Acidobacteria, the higher relative abundance of most subphylum groups, including DA052 and KF-JG30-18, was found to be linked to aluminum, iron and manganese (*p* ≤ 0.05 [corrected according to Benjamini and Hochberg, 1995]). DA111 (Alphaproteobacteria), but also JG30-KF-CM66 and KD4-96 (Chloroflexi), showed a strong correlation with aluminum and iron (*p* ≤ 0.05). The Actinobacteria showed high correlations with all recorded environmental parameters. The significance of these correlations, however, has to be considered low, given that members of this phylum showed no distinct distribution pattern between the different sampling sites (Figures 1A,B). The high aluminum content at the three slag deposition sites (RH1-3) (Figure 2A) and the relatively low pH (Figure 2B) may point to a high bioavailability of toxic aluminum species (Supplementary Figures 7, 8).

### 3.3. Reconstruction of metagenome-assembled genomes

**Figure 3:**
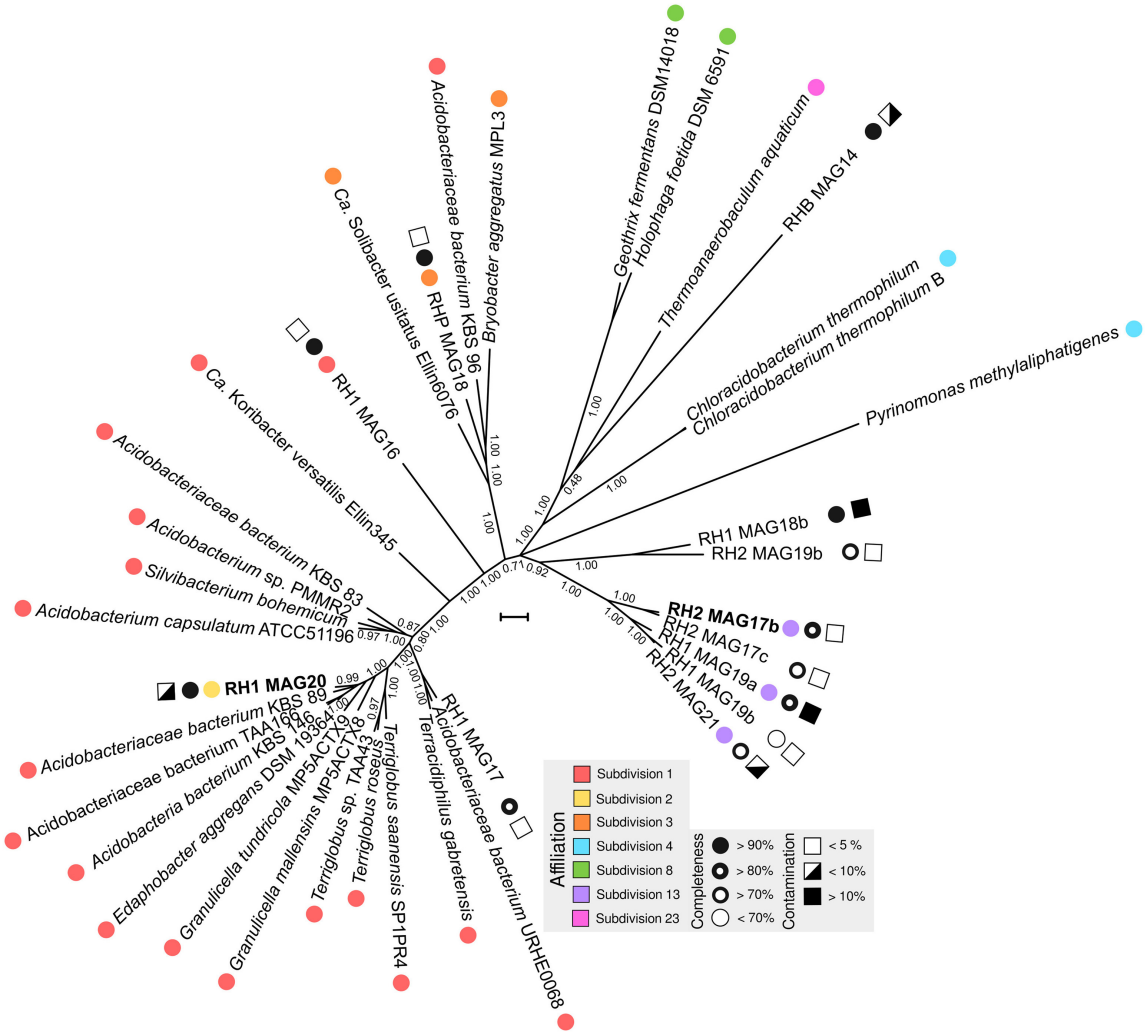
Phylogenetic tree of MAGs. Available Acidobacteria genomes of known phylogenetic affiliation were retrieved from the NCBI genome repository. Phylogenetic treeing was based on a concatenated alignment of 31 single-copy marker genes using AMPHORA (v. 2) (Wu and Scott, 2012). Using FASTTREE (v. 2.1.3) (Price et al., 2010), a maximum-likelihood tree was calculated applying the WAG model (Whelan and Goldman, 2001). Nearest neighbor interchange and the CAT approximation were used to optimize tree topology and to consider evolutionary rate heterogeneity (Stamatakis, 2014). Colored circles indicate the taxonomic affiliation based on available 16S rRNA gene sequences. Black-white spheres and squares show the degree of completeness and contamination, which was derived from the presence/absence of single-copy marker genes and their actual copy number in the MAGs (Parks et al., 2015). MAGs analyzed in more detail are highlighted in bold. The scale bar indicates 0.1 substitutions per amino acid position.

An overview of processed metagenome datasets is given in Supplementary Table 3. Due to the co-dominance of yet-uncharacterized Acidobacteria groups on subphylum level (Figure 1), we were interested in elucidating both their metabolic potential and their specific adaptations favoring their persistence in slag deposits. Application of a two-step binning strategy (Supplementary Figure 4) allowed us to reconstruct 13 Acidobacteria MAGs (RH1: 6, RH2: 5, RHP: 2) (Supplementary Table 4). A major proportion of MAGs was of high quality in terms of completeness (8 out of 13, >80% complete) and contamination (9 out of 13, <10% contaminated) (Figure 3). Based on treeing analysis of a concatenated alignment of 31 single-copy marker genes, a group of 5 MAGs (RH2 MAG 17b, RH2 MAG 17c, RH1 MAG 19a, RH1 MAG 19b, and RH2 MAG21) had an uncertain affiliation with no close relationship to any described genus. 16S rRNA gene sequences could be identified for 6 MAGs. Based on identified 16S rRNA gene sequences it was possible to link two genomes to uncharacterized taxonomic groups (DA052 and KF-JG30-18) that were found to be dominant in the bacterial community profiles (Figure 1B). These two genomes were analyzed in greater detail (RH1 MAG 20 [completeness: 94%]: DA052 [Acidobacteria, Subdivision 2]; RH2 MAG 17b [completeness: 89%]: KF-JG30-18 [Acidobacteria, Subdivision 13]). The focus of our genome analysis was on carbon utilization capabilities and adaptations favoring colonization of Red Hill slag deposits.

### 3.4. MAG analysis of KF-JG30-18 and DA052

**Figure 4:**
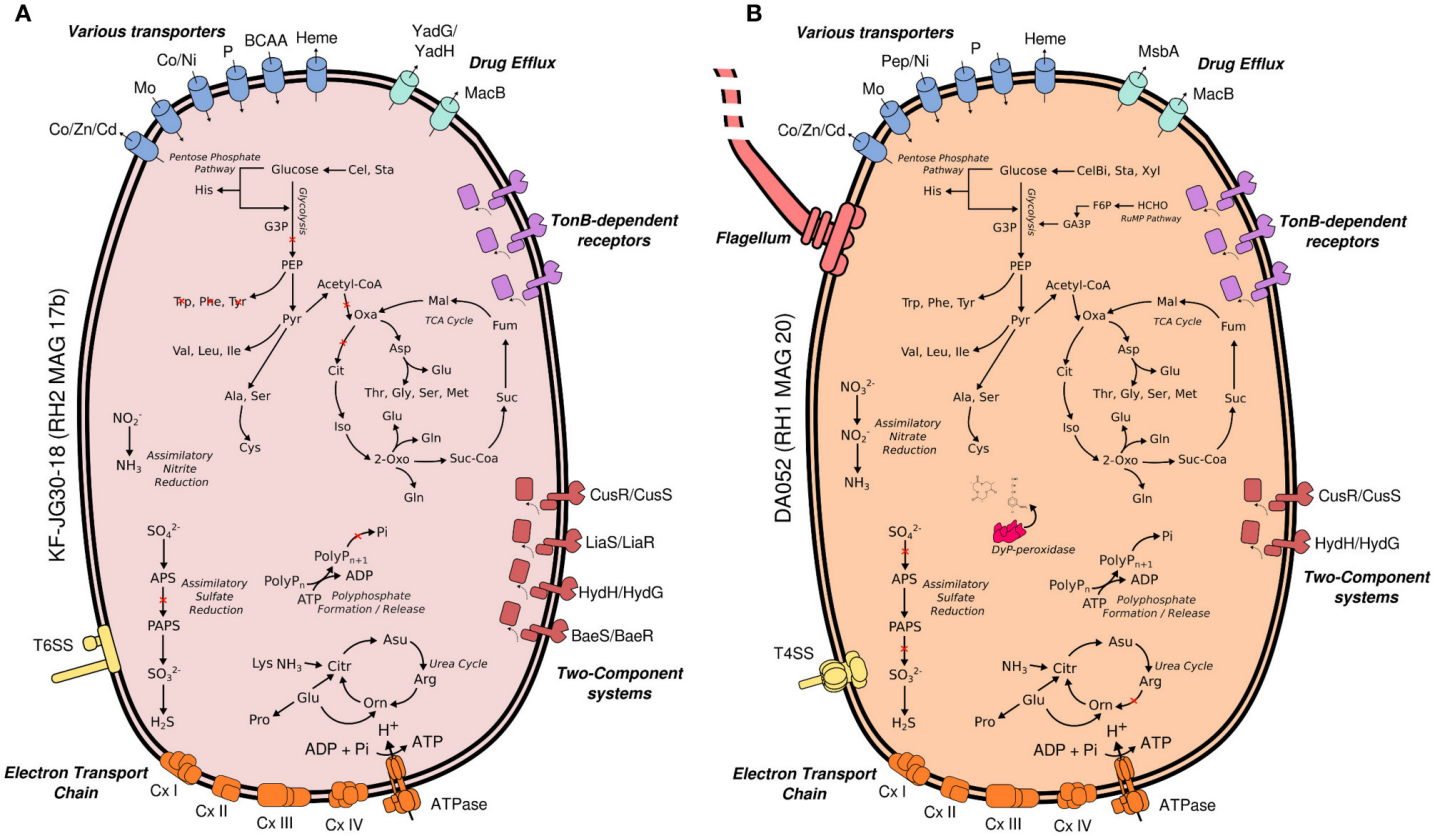
Metabolic traits of KF-JG30-18 **(A)** and DA052 **(B)** as derived from the genetic potential of RH2 MAG 17b and RH1 MAG20. Functional annotations are based on PROKKA (Seemann, 2014) and RAST (Aziz et al., 2008; Overbeek et al., 2014). Both annotation results were thoroughly validated against each other. Pathways were reconstructed by UBLAST (Edgar, 2010) searches of predicted coding genes against NCBI nr. Predicted genes were additionally classified against the KEGG reference database using KEGGMAPPER. Red crosses indicate missing genes in the respective pathways. Co/Zn/Cd (cobalt/zinc/cadmium), Czc efflux pumps; Mo, molybdate; Ni, nickel; BCAA, branched-chain amino acids; YadG/YadH, uncharacterized ABC transporter; MsbA + MdlB, multi-drug resistant ABC transporter; MacB + YddA, macrolide-binding ABC transporter; CusS/CusR, two-component system sensing extracellular copper; HydH/HydG, two-component system sensing extracellular metal ions; LiaS/LiaR, two-component system sensing cell envelope stress; BaeS/BaeR, two-component system involved in antibiotic sensing; TXSS, type X secretion system; CxI-IV, complex I to IV of the respiratory chain; G3P, glycerate 3-phosphate; PEP, phosphoenolpyruvate; Citr, citrulline; Orn, ornithine; PolyP, polyphosphate; Oxa, oxaloacetate; Mal, malate; Fum, fumarate; Suc, succinate; Suc-CoA, succinyl-CoA; 2 Oxo, 2-oxoglutarate; Iso, isocitrate; Cit, citrate; APS, adenosine phosphosulfate; PAPS, phosphoadenosine phosphosulfate; Cel, cellulose; CelB, cellubiose; Sta, starch; Xyl, xylan; F6P, fructose 6-Phosphate; GA3P, glyceroaldehyde 3-phosphate; G3P, glycerol-3-phosphate; RuMP, ribulose monophosphate pathway; Dyp, dye-decolorizing peroxidases.

Both Acidobacteria MAGs encode key pathways for central carbohydrate metabolism, most pathways for the synthesis of proteinogenic amino acids and complete respiratory chains, thereby suggesting a heterotrophic lifestyle (Figure 4). In case of KF-JG30-18, the failure to detect the complete gene sets encoding glycolysis and TCA cycle can be attributed to the incompleteness of its MAG (Supplementary Table 4).

#### 3.4.1. KF-JG30-18

RH2 MAG 17b showed a total length of 2.8 Mb with a GC content of 58% (Table 1). The KF-JG30-18 representative has the genetic potential to utilize the polysaccharides cellulose and starch as carbon source and nitrite as additional nitrogen source (Figure 4A). While no sulfate adenylkinase (EC 2.7.1.25) gene is present, KF-JG30-18 carries all the other genes for assimilatory sulfate reduction. The presence of genes encoding CusS/CusR and HydH/HydG two-component systems enables sensing of extracellular metal cations. BaeS/BaeR and LiaS/LiaR two-component systems allow detection of cell envelope stress. KF-JG30-18 possesses 11 genes linked to CzcCBA efflux systems, which belong to the multifunctional family of type VI secretion systems. It also contains various transporter genes including those for molybdate, cobalt/nickel, branched-chain amino acid uptake systems, and heme export systems. In addition, genes encoding high-affinity phosphate uptake systems and genes encoding enzymes facilitating polyphosphate formation were detected for KF-JG30-18. Finally, two CRISPR arrays were identified (Table 1).

**Table 1:** Characteristics of RH1 MAG 20 (DA052) and RH2 MAG 17b (KF-JG30-18).

	**RH1 MAG20 (DA052)**	**RH2 MAG 17b (KF-JG30-18)**
Total length [bp]	4,885,294	2,798,462
No. of recruited reads	404,720 406,649	254,272 254,736
Coverage	18	18
No. contigs	760	169
N50 [bp]	58,735	25,655
GC content [%]	59	58
Completeness [%]	93.97	89.39
Contamination [%]	5.15	2.14
Classification^*^	Nearly complete genome with medium contamination	Substantial genome with low contamination
No. of coding genes	5,311	2,822
No. of rRNA operons	1	1
No. of tRNAs	44	35
Mobile elements^**^	29	3
CRISPR arrays	–	2
No. of encoded Czc efflux pumps	29	11
No. of encoded Acriflavine resistance proteins	5	6

*Recruited reads refer to the number of reads incorporated during the assembly of respective contigs. Numbers represent forward and reverse reads. Coverage was determined by determining the average coverage of genome bin contigs. Genome completeness and contamination have been assessed using CHECKM (Parks et al., 2015). The number of coding genes, rRNA operons, tRNAs, mobile elements, CRISPR/CAS repeats and repeat regions is given based on genome annotations done with PROKKA (v. 1.11) (Seemann, 2014) and RAST (v. 2.0) (Aziz et al., 2008; Overbeek et al., 2014) followed by manual refining*.

* *According to Parks et al. (2015)*;

** *transposons and insertion sequences*.

#### 3.4.2. DA052

The RH1 MAG 20 genome was substantially bigger in comparison to KF-JG30-18 and showed a total length of 4.9 Mb featuring a GC content of 59% (Table 1). In contrast to KF-JG30-18, the DA052 representative (RH1 MAG 20) has the capacity to express the ribulose monophosphate pathway, suggesting the potential to metabolize C1 compounds by formaldehyde fixation. The RH1 MAG 20 also possesses genes involved in the breakdown of cellobiose, starch and xylan. In addition, genes that encode dye-decolorizing peroxidases were detected. Like KF-JG30-18, DA052 has the potential to utilize oxidized nitrogen species such as nitrite and nitrate as additional nitrogen sources (Figure 4B). Albeit no adenyltransferase (EC 2.7.7.4) gene could be identified, DA052 is likely able to carry out assimilatory sulfate reduction. The complete set of genes required for flagellar motility and type IV secretion was identified in RH1 MAG 20. It also encodes high-affinity phosphate uptake systems, is equipped with the potential to form and release polyphosphate, and contains multiple genes that code for phosphate-binding subunits. Genes involved in sensing extracellular metal cations were detected, such as those encoding CusS/CusR and HydH/HydG two-component systems. DA052 contains even more genes associated with CzcCBA efflux systems (29 genes) than KF-JG30-18 (11). Worth mentioning is also the high number of transposase genes and genes encoding mobile element proteins (29) (Table 1).

## 4. Discussion

The slag samples contained high amounts of iron and sulfur (Figure 2A), presumably in mineral form. This was unexpected given the age (150 years) and leached nature of the slags. Assuming a continuous exposure to oxygen and humidity, an oxidation of present sulfide-mineral components was anticipated. Sulfide-rich mine tailings show a zonation over time: a sulfide-depleted zone; an active, transitory oxidation zone; and a primary zone representing the original conditions (Diaby et al., 2007; Tan et al., 2008; Huang et al., 2011). In our study, the slags originated from soft coal with a high content of pyrite. Organic carbon in soft coal and, as a consequence, in the studied slags primarily consists of humic and fulvic acids (Peuravuori et al., 2006). Humic acids were repeatedly reported to be efficient in pyrite passivation (Lalvani et al., 1996; Belzile et al., 1997; Chen et al., 1999), either by chelating released ferric iron with their negatively charged functional (carboxylic, phenolic) groups or by direct pyrite adsorption. Both processes may explain why the oxidation of sulfide minerals in the slag deposits was mitigated. This low oxidation level clearly differentiates the Red Hill slag deposits from mine tailings and other AMD environments.

Aluminum was found to be a major driver of bacterial community structure in the leached slags (Figures 2B–D). The high aluminum content highlights the inefficiency of the early-industrial leaching process. Given the low pH (3.4–3.6) of the slags, aluminum was likely present as cations (Supplementary Figure 7). Cationic aluminum is highly toxic because of potential interferences with ATP metabolism, lipid metabolism and membrane transport processes (Supplementary Figure 8). In soils, aluminum toxicity can be mitigated by clay minerals and organic acids, which act as a buffer system due to their cation exchange capacity (Turpault et al., 1996).

The chelating capabilities of humic and fulvic acids may lead to the immobilization of aluminum cations as described above for iron-sulfur minerals. Mohan and Chander (2006) showed that brown coal is an efficient adsorbent for metal cations in AMD discharges. Given a passivation of iron-sulfur minerals and aluminum, their role as drivers for bacterial community structure can be considered indirect, albeit toxic effects of aluminum due to minor releases may occur. Previous research had revealed that the retention of metal cations can alter the accessibility and utilizability of humic and fulvic acids as potential carbon sources for microbes (Jones and Edwards, 1998; Jones et al., 2003). Considering that humic and fulvic acids are naturally rather inert and recalcitrant carbon sources (Kirk and Farrell, 1987), metal complexation would increase the supposed oligotrophic nature of the studied slag deposits.

In the leached slags, both microbial biomass and bacterial cell numbers were significantly lower than in the undisturbed forest soil (Supplementary Figure 5), but in the range previously determined for sulfidic mine tailings (Korehi et al., 2014). Five yet-uncultured subphylum groups were identified to be dominant in the slag deposits. These belonged to Acidobacteria, Actinobacteria, Alphaproteobacteria, and Chloroflexi (Figures 1A,B). None of the five groups have previously been reported to be highly abundant in AMD systems. Their first detection was in peatlands (DA052, DA111, TM214) (Rheims et al., 1996; Felske et al., 1998) and uranium waste piles (KF-JG30-18, JG37-AG-4, DA111) (Selenska-Pobell et al., 2001; Selenska-Pobell, 2002). Phylogenetic analysis of nearly complete 16S rRNA gene sequences also revealed relationships to populations present in acidic, metal-rich, and contaminated soil environments (Supplementary Table 2). In accordance with the geochemical differences discussed above, non-occurrence of bacteria known to be involved in iron- and sulfur-cycling again clearly differentiates the slag deposits from AMD systems and mine tailings.

Given that cultured representatives are not yet available for the dominant groups identified in the Red Hill slag deposits, their metabolic capabilities remained elusive. Therefore, high abundances of Acidobacteria subdivisions 2 (DA052) and 13 (KF-JG30-18) prompted us to conduct a metagenomic approach in order get first insights into their genetic potential. Acidobacteria are known to be abundant members of terrestrial microbiomes (Janssen et al., 2002; Sait et al., 2006) and to be phylogenetically highly diverse (Barns et al., 2007). A total of 22 genera have been described, which cover only 8 of 26 subdivisions proposed until today. Genome sequences are available for members of 5 subdivisions (1, 3, 4, 8, and 23; Kielak et al., 2016). Acidobacteria are believed to have an oligotrophic (K-strategist) lifestyle. This perception, however, may be biased due to the low number of acidobacterial isolates of which most belong to subdivision 1. Members of subdivisions 2 and 13 have repeatedly been detected in higher abundances in U-contaminated environments (Selenska-Pobell et al., 2001; Barns et al., 2007). The occurrence of subdivision 2 populations in Amazonian forest soils was correlated to the microbial availability of CO_2_, Fe, and Al^3+^. Thus, it was hypothesized that members of subdivision 2 are tolerant to aluminum (Navarrete et al., 2013; Catão et al., 2014). Adaptation and tolerance to Al^3+^ could also be one major reason for the high relative abundance of DA052 in our sampling sites, especially in RH1. Amazonian forest soils are commonly rich in biochar whose chemical characteristics are remotely similar to those of studied soft coal slags. However, a recent study of biochar microbiomes and metagenomes revealed a rather low abundance of Acidobacteria (Noyce et al., 2016). Given the limited knowledge of yet uncharacterized Acidobacteria groups, the MAGs obtained for subdivisions 2 (DA052) and 13 (KF-JG30-18) allowed us to infer valuable information on the physiology and metabolic potential of these two Acidobacteria subdivisions.

As of November 2016, 27 genome sequences were available for Acidobacteria, with most of them being affiliated with subdivision 1 Acidobacteria. This number has recently more than doubled by deep metagenomic sequencing of an aquifer system (Anantharaman et al., 2016). However, to the best of our knowledge, no genomes of Acidobacteria subdivisions 2 and 13 have yet been reported, highlighting the novelty of the draft genomes reconstructed for DA052 (RH1 MAG20) and KF-JG30-18 (RH2 MAG 17b). The genome size of DA052 is comparable to those of subdivision 1 Acidobacteria such as *Terriglobus roseus* (4.9 Mb, 60% GC) (Rawat et al., 2014). The KF-JG30-18 genome is comparably small, being in the range of the only subdivision 23 genome (*Thermoanaerobaculum aquaticum* 2.7 Mb)(Stamps et al., 2014). Apparently, members of DA052 and KF-JG30-18 are able to utilize polysaccharides such as cellulose and xylan (Figure 4). Comparative genomics suggests that the ability of polysaccharide breakdown is widely distributed among the Acidobacteria (Kielak et al., 2016), but to date experimental evidence for polysaccharide utilization was obtained only for members of subdivisions 1, 3, and 4 (Dedysh et al., 2012; Pascual et al., 2015; Garcia-Fraile et al., 2016; Jiang et al., 2016; Lladó et al., 2016). Xylan degradation appears to be a rather common metabolic trait of the Acidobacteria, while cellulose breakdown has been shown only for *Telmatobacter bradus* (Pankratov et al., 2008). Plant cell wall constituents such as cellulose or xylan are commonly identified in soft coal in varying quantities dependent on the degree of coalification (del Río et al., 1994; Rumpel et al., 1998). The DA052 draft genome RH1 MAG20 contains all the genes required for formaldehyde fixation via the cyclic ribulose monophosphate pathway (Figure 4). Thus, members of DA052 may be able to utilize C1 compounds as carbon source. These are present in coal as methyl- and methoxyl-groups or as formate (Stafford, 1988; Stout et al., 1988; Hatcher and Clifford, 1997). A survey of the available Acidobacteria genomes revealed key genes of the ribulose monophosphate pathway (3-hexulose-6-phosphate synthase and 6-phospho-3-hexuloisomerase) in *Edaphobacter aggregans* and *Terriglobus roseus*, but both organisms have not yet been shown to utilize C1 compounds (Eichorst et al., 2007; Koch et al., 2008). In the DA052 representative, we also identified the genetic potential to produce lignin-degrading dye-decolorizing peroxidases. This family of enzymes is of increasing interest as it represents the bacterial counterpart of lignin-cleaving peroxidases and laccases known from fungi (de Gonzalo et al., 2016). The ability to utilize rather inert, lignin-derived carbon sources would give DA052 a selective advantage as lignin-derived carbon is assumed to be abundantly present in the studied slags.

Although most aluminum in the Red Hill slag deposits is assumed to be immobilized by humic and fulvic acids, some bioavailability cannot be excluded. Both draft genomes, DA052 and KF-JG30-18, contain genes encoding high-affinity phosphate uptake systems, while the potential to form and release polyphosphate was detected specifically for DA052. The intracellular cleavage of polyphosphate and release of phosphate moieties was described for bacteria and archaea (Remonsellez et al., 2006; Rao et al., 2009; Navarro et al., 2013) as a measure to inactivate toxic metal cations by precipitation. Since inorganic phosphate has manifold roles in cellular metabolism, the sole presence of these genes cannot be exclusively attributed to metal resistance. The draft genomes of both DA052 and KF-JG30-18, however, harbor an unexpectedly high number of genes encoding Czc efflux pumps (Figure 4). These mediate resistance to cobalt, zinc, and cadmium (Nies, 1995; Chen et al., 2015). Using InterPro and PFAM, a closer inspection of the encoded proteins revealed that 6 and 5 sequences for, respectively, KF-JG30-18 and DA052 are closely related to acriflavine resistance proteins, which, like Czc efflux pumps, belong to the resistance/nodulation/division superfamily of solute transporters (Table 1). Acriflavine resistance proteins are multi-drug efflux systems with a tremendously broad substrate range, including different classes of antibiotics, detergents and small organic molecules (Blair and Piddock, 2009; Pos, 2009). Acriflavine was first extracted from smoldered coal tar by precipitation with diluted sulfuric acid, a procedure that is reminiscent of the early industrial mineral leaching from soft coal (Supplementary Figure 1). Paul Ehrlich found acriflavine to be bacteriocidal (Wainwright, 2001; Kumar et al., 2012) and intercalation into DNA was identified as its mode of action (Lerman, 1961). It has been shown for iron-oxidizing bacteria that the number of acriflavine resistance genes can have a strong effect on their fitness when being exposed to metals, metalloids, or hazardous organic compounds (Emerson et al., 2013). It is thus tempting to hypothesize that the presence of such substances in the leached slags selects for acriflavine-tolerant bacteria. In consequence, the presence of multiple genes encoding acriflavine resistance proteins could thus represent a competitive advantage, favoring the colonization of studied slags by DA052 and KF-JG30-18.

In summary, our analyses revealed that the microbiology of the studied slags greatly differs from commonly studied AMD and mine tailing environments. The unusual geochemistry, including the proposed mitigation of mineral oxidation and subsequent metal release, increases the oligotrophic nature of the Red Hill slag deposits. Members of the elusive Acidobacteria subdivisions 2 (DA052) and 13 (KF-JG30-18) showed a dominant occurrence not observed in any other previously studied environment. Metagenome-assembled genomes allowed us to identify broad carbon utilization capabilities and, more intriguingly, pronounced metal and xenobiotic detoxification mechanisms. These may explain the dominant occurrence of DA052 and KF-JG30-18. Our findings call for further research into the microbiology of the Red Hill slag deposits, in particular for activity-centered approaches such as metatranscriptomics and -proteomics that allow to link both genome coding potential and actual metabolic activity of identified groups in this unique, early-industrial, man-made habitat.

## Author contributions

CW and WL designed research. CW carried out research, analyzed data, wrote code. CW and WL wrote the manuscript together.

### Conflict of interest statement

The authors declare that the research was conducted in the absence of any commercial or financial relationships that could be construed as a potential conflict of interest.
