# Supplementary material for: Unexpected Dominance of Elusive Acidobacteria in Early Industrial Soft Coal Slags
Source: Front Microbiol. 2017 Jun 8;8:1023. doi: 10.3389/fmicb.2017.01023 (PMC5462947; doi:10.3389/fmicb.2017.01023)
Supplement: Supplementary file 1 [file Presentation1.pdf]

Supplementary Material

Unexpected dominance of elusive Acidobacteria in early industrial soft coal slags

Carl-Eric Wegner [1,2] **and Werner Liesack** [1,*]

[1] *Max Planck Institute for Terrestrial Microbiology, Department of Biogeochemistry, Karl-von-Frisch-Str. 10, 35043 Marburg, Germany*
[2] *Friedrich Schiller University Jena, Institute of Ecology, Aquatic Geomicrobiology, Dornburger Str. 159, 07749 Jena, Germany*

Correspondence*:
Werner Liesack
liesack@mpi-marburg.mpg.de

1 SUPPLEMENTARY METHODS

1.1 Location, sampling, site description

Sampling was carried out at a slag deposition site belonging to former alum factories in the south-east of Bonn (Northrhine-Westphalia, Germany). The slag is characterized by a distinct red color (Supplementary Figure 1) and the sampling site shows a strongly reduced vegetation. In the 19th century, soft coal mining in this area was carried out due to the alum content of the coal. Mined coal was roasted, the resulting ash leached and the leachate boiled yielding crystalline alum as the end product (Supplementary Figure 1) (Falk, 2002). The main by-product of the leaching process was slag, which was dumped without precautions or remediation in the near surrounding. Our study sites date back to the 1860s. After sampling, samples were kept at ambient temperature, transported to the laboratory and homogenized within 4 hours. Aliquots (0.5 g) of slag material were subsampled in 2 mL screwcap tubes, flash-frozen and stored at -20 °C for downstream molecular analyses. Samples for CHNS (carbon, nitrogen, hydrogen and sulfur) analysis were ground and dried for 48 hours at 65 °C. Aliquots (10 g) for elemental analysis were kept at room temperature. Samples for BTEX (benzene, toluene, ethylbenzene and xylene) and PAH (polycyclic aromatic hydrocarbons) analyses were freshly taken in June 2015. BTEX samples (2.5 g of slag) were kept in brown, methanol-filled vessels. Samples for PAH determination (50 g) were kept in glas jars.

1.2 Analysis of geochemical parameters

The total content of CHNS was determined by high temperature combustion using a varioMICRO elemental analyzer (Elementar Analysensysteme, Hanau, Germany). For elemental analysis, slag samples were extracted with concentrated HCl. The contents of aluminium, calcium, iron, manganese and phosphate were determined based on inductively coupled plasma mass spectrometry (ICP-MS). Heavy metal contents were assessed by atomic absorption spectroscopy (AAS). The presence of PAH and BTEX was examined by Eurofins Umwelt West GmbH (Wesseling, Germany).

1.3 Reaction conditions for 16S rRNA gene-targeted PCR

Each PCR reaction (50 µL) contained: 10 µL GoTaq FlexiBuffer (5x) (Promega, Mannheim, Germany), 1 µL dNTPs (10 mM each), 4 µL $MgCl_2$ (25 mM), 0.25 µL GoTaq Polymerase (Promega), 1 µL of each primer (20 pmol/µL), 31.75 µL of PCR-grade water and 1 µL of template DNA. The thermal profile was as follows: 95 °C for 5 min., 25 cycles of 95 °C for 40 s, 55 °C for 2 min. and 72 °C for 1 min. followed by a final extension step of 7 min. at 72 °C.

1.4 Quality control and initial processing of amplicon sequencing data

General sequence characteristics were assessed using PRINSEQ (v. 0.20.4) (Schmieder and Edwards, 2011). Residual adaptor sequences were trimmed applying CUTADAPT (v. 1.7) (Martin, 2010). Paired-end sequences were assembled and quality-controlled with USEARCH (v. 7.0) (Edgar, 2010) using the implemented -fastq_mergepairs (-fastq_minmergelen = expected amplicon sizes) and fastq_filter (-fastq_maxee = 0.5) functions. Potential chimeric sequences were identified with UCHIME (v. 4.2.40) (Edgar et al., 2011).

1.5 Two-step binning and reconstruction of metagenome-assembled genomes

A two-step binning procedure was used to obtain metagenome-assembled genomes (MAGs). In the first binning step, VIZBIN (Laczny et al., 2015) was applied to inspect assembled contigs ($\geq$ 1000 bp). K-mer based ordinations were compressed using non-linear dimension reduction applying Barnes-Hut stochastic neighbor embedding. Clusters of contigs having the same taxonomic identity were extracted. In the second binning step, GC-content and coverage information were used to resolve potential heterogeneities within recovered clusters of contigs (Supplementary Figure 4). Sequences used for the assembly of the respective contigs were recruited using BBMAP and subjected to reassembly. Reassemblies were optimized using MINIMUS2 implemented in the amos software suite (v. 3.1.0, http://amos.sourceforge.net/wiki/index.php/AMOS). The resulting subsets of contigs were considered putative genome bins.

1.6 Statistical analyses

Significant differences between sampling sites (geochemical parameters, bacterial community profiles) were analysed by one-way ANOVA (analysis of variance), using Tukey-Kramer's test for post-hoc analysis (Tukey, 1949) and applying the correction for multiple comparisons according to Bonferroni. Multiple regression analyses to determine links between particular bacterial phyla and geochemical parameters of interest were carried out with log-transformed data. Results from these analyses were validated by Mantel tests for individual environmental parameters. Single correlation analyses between selected environmental parameters and bacterial species richness were done by calculating Pearson correlation coefficients. Correlations between either individual environmental parameters or sets of environmental parameters and microbial community structure were determined by relating environmental parameters to calculated Jensen-Shannon divergences using the anosim and bioenv functions implemented in vegan (Oksanen et al., 2016). Kendall's tau coefficients were used to link the occurrence of specific taxa within phyla of interest to selected environmental parameters. Determined p-values were corrected for multiple comparisons applying the method introduced by Benjamini and Hochberg (Benjamini and Hochberg, 1995).

2 FIGURES

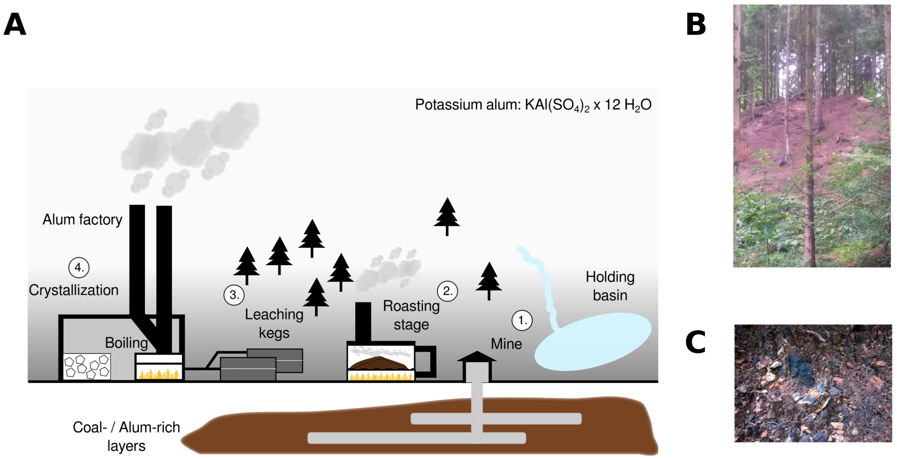

Supplementary Figure 1. Early industrial alum leaching from soft coal. Alum-leaching from alum-rich soft coal was a four-step process (A). Alum-rich coal was mined (1), crushed, ground to smaller pieces and smoldered (2) yielding ash enriched in alum. The ash was leached out using water (3) and the leachate was subsequently boiled yielding crystalline alum (4). One ton of alum-rich coal yielded on average 50 kg of crystalline alum, and 300-400 kg of leached slag as by-product (Falk, 2002). Leached slag was dumped in the surrounding of former alum factories without any precautions or remediation. Slag deposits in the surrounding of former alum factories feature a distinct red colour and they show a strongly reduced vegetation (B). A closer inspection of dumped slag revealed the presence of combustion remnants and even fully intact pieces of coal (C).

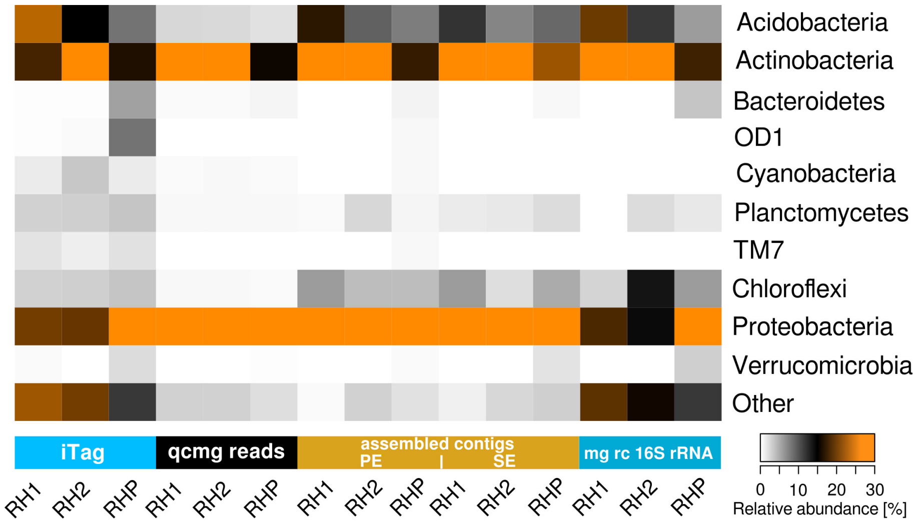

Supplementary Figure 2. Phylum-level taxonomic profiles of metagenome datasets at different stages of analysis in comparison to community profiles derived from amplicon sequencing. The taxonomic affiliation of quality-controlled metagenome sequences (qcmg reads) was determined using KRAKEN (Wood and Salzberg, 2014). Full-length 16S rRNA sequences (mg rc 16S rRNA) were reconstructed with EMIRGE (Miller et al., 2011). Starting either from single-end (SE) or paired-end (PE) reads, assembled contigs were taxonomically binned applying PHYLOPYTHIA S+ (Gregor et al., 2016).

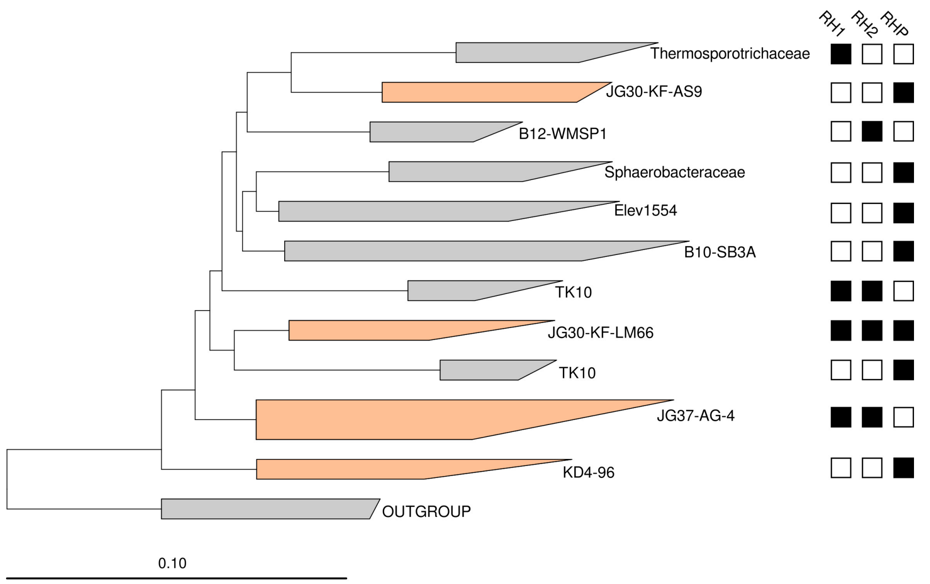

Supplementary Figure 3. Phylogenetic affiliation of Chloroflexi full-length 16S rRNA gene sequences. Sequences were reconstructed using EMIRGE (Miller et al., 2011). Orange-shaded clades refer to uncharacterized groups that have been particularly abundant in iTag sequencing. The presence / absence chart (RH1, RH2, RHP) indicates agreement with the iTag sequencing data, highlighting that the reconstruction of full-length 16S rRNA gene sequences was possible for the respective dataset. The NJ phylogenetic tree was calculated using ARB (v. 6.0.2) (Ludwig et al., 2004). The scale bar refers to 0.1 changes per nucleotide; NJ = neighbour-joining.

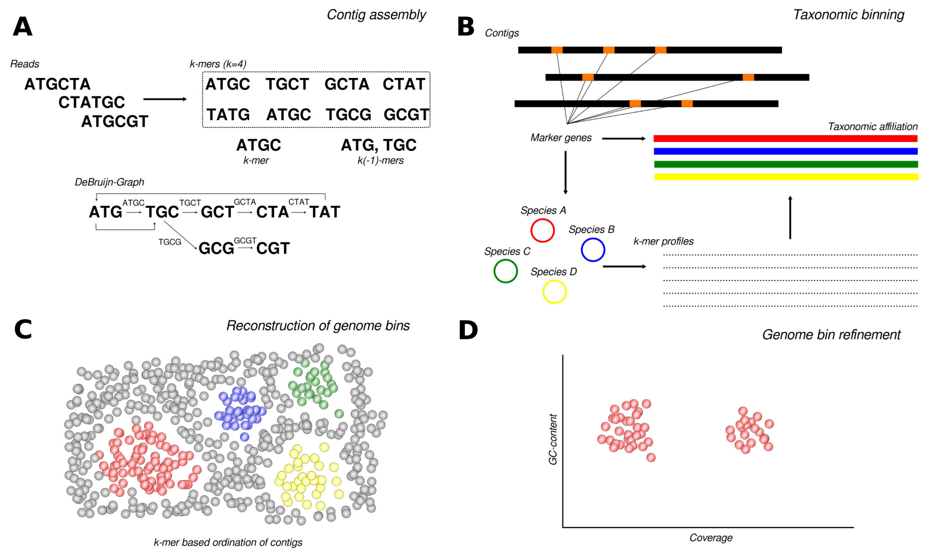

Supplementary Figure 4. Reconstruction of metagenome-assembled genomes (MAGs). A two-step binning approach was applied to recover putative genomes. Quality-controlled data was assembled using megahit (Li et al., 2014) as iterative DeBruijn graph-based assembler applying a k-mer range from 33 – 73 (step-size 10) (A). Contigs were taxonomically binned using both single-copy marker genes present on contigs and k-mer profiles applying PHYLOPYTHIA S+ (Gregor et al., 2016) (B). In the first binning step, VIZBIN (Laczny et al., 2015) was applied to inspect assembled contigs. K-mer based ordinations were compressed using non-linear dimension reduction applying Barnes-Hut stochastic neighbor embedding. Clusters of contigs having the same taxonomic identity were extracted (C). In the second binning step, GC-content and coverage information were used to resolve potential heterogeneities within recovered clusters of contigs (D).

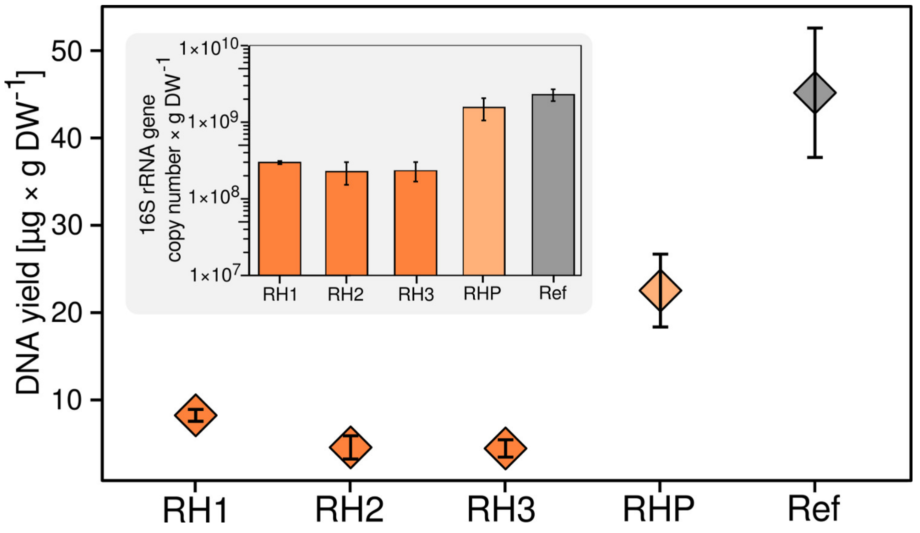

Supplementary Figure 5. Recovery of microbial biomass. Bacterial 16S rRNA gene copy numbers were determined using quantitative PCR (n = 3), as outlined in the main text. The yield of total DNA from slag (RH1-RH3), pond (RHP) and forest soil (Ref) samples was estimated (n=3) by determining the dry weight of samples used for DNA extraction and relating the dry weight to the recovered amount of total DNA.

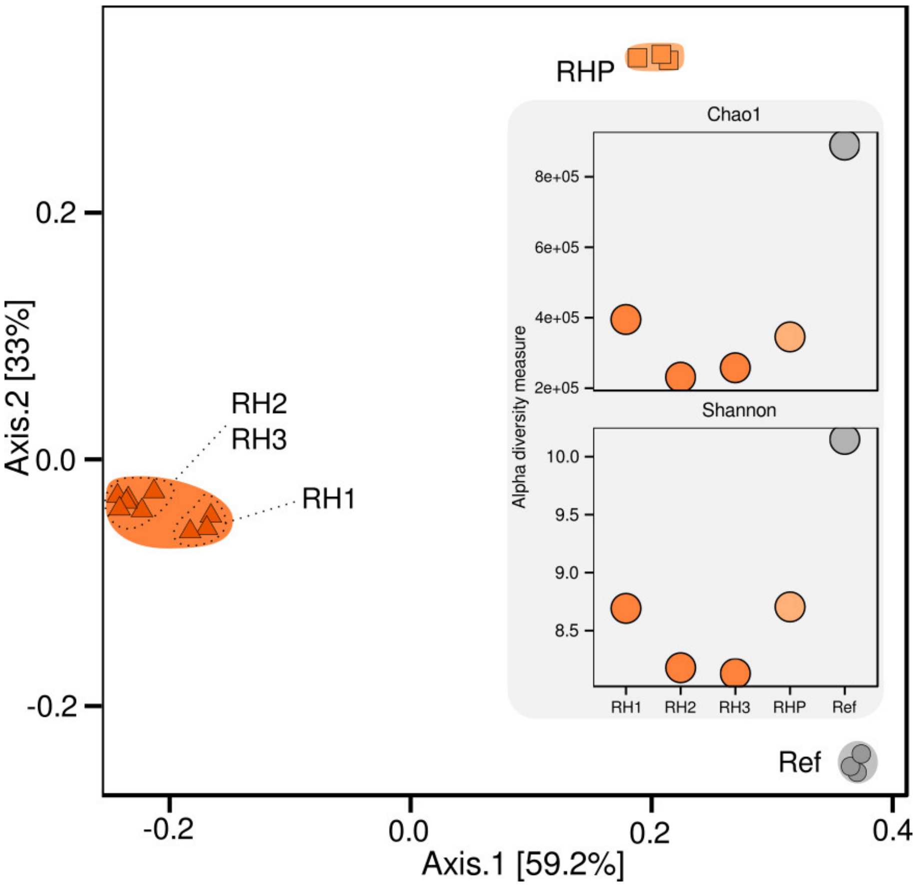

Supplementary Figure 6. Alpha- and beta-diversity analyses of slag bacterial communities. Chao1 and Shannon indices were calculated as measures for estimated bacterial species richness and evenness. Beta-diversity was assessed based on Jensen-Shannon divergences (Lin, 1991).

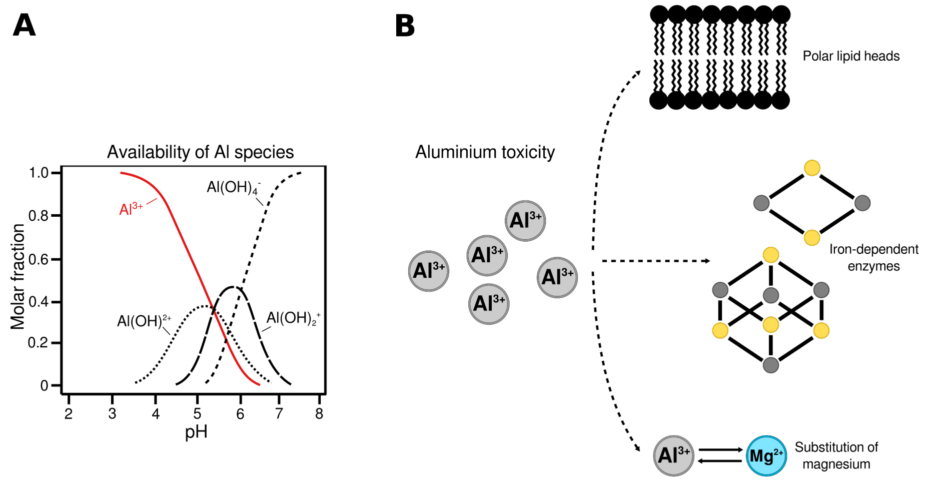

Supplementary Figure 7. The pH dependence of aluminium species and the toxicity of bioavailable aluminium. The availability of aluminium species is strongly related to pH (Piña and Cervantes, 1996). At neutral or near neutral pH, aluminium is mostly present in form of oxides, which are not bioavailable. With decreasing pH, aluminium is increasingly present in its bioavailable and toxic form. Aluminium toxicity is mediated by a couple of mechanisms including, for instance, the interaction with the polar heads of membrane lipids and polar groups of membrane proteins. Both impair membrane transport processes (Schroeder, 1988; Zambenedetti et al., 1994) . Aluminium cations are known to have orders of magnitude higher affinity for ATP than magnesium (affinity of Al^{3+} higher by a factor of 10^7 (Macdonald and Bruce Martin, 1988)), which can have fatal effects on ATP metabolism. Due to similar chemical properties aluminium can replace iron in iron-dependent enzymes of which many are found in the respiratory chain and the tricarboxylic acid cycle. Considering that aluminium is not redox-active, the replacement of iron with aluminium renders corresponding enzymes inactive.

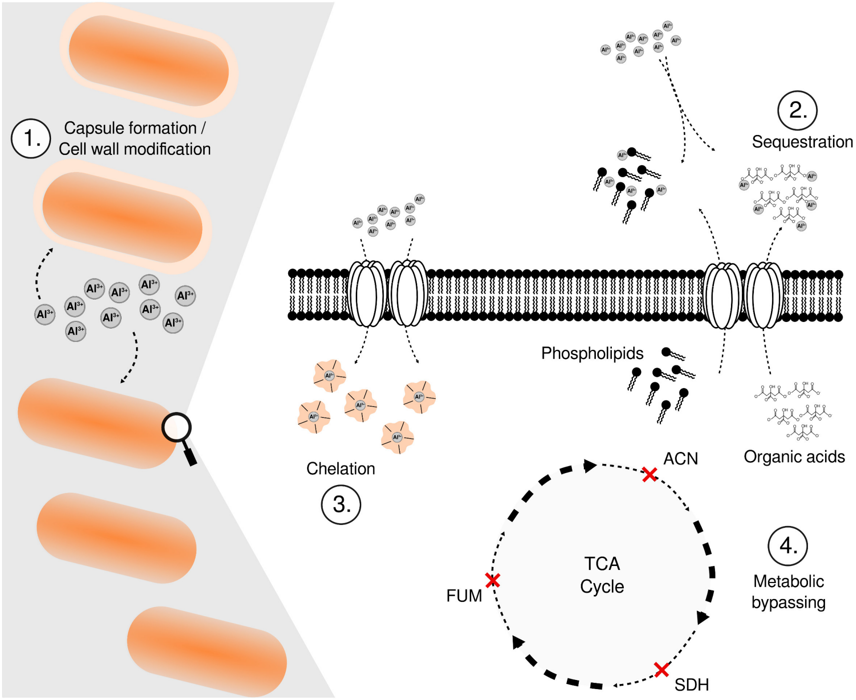

Supplementary Figure 8. Potential aluminium tolerance / resistance mechanisms. Exemplary mechanisms include cell wall modifications, for instance the incorporation of glycosphingolipid instead of phospholipids and capsule formation (1). Both mechanisms prevent / restrict the uptake of aluminium cations. Chelation and sequestration by specific binding proteins, phospholipids, organis acids, amino acids and phosphate are additional mechanisms to cope with aluminium toxicity (2, 3). The fatal effect of aluminium on iron-dependent enzymes (e.g. various enzymes of the respiratory chain and the tricarboxylic acid cycle) makes metabolic bypassing necessary, meaning the usage of analogous, iron-free enzymes (4). Finally, general stress responses, such as efflux systems and environmental sensing, can play a role (Piña and Cervantes, 1996). ACN = aconitase, SDH = succinate dehydrogenase, FUM = fumarase (all three are examples of iron-dependent enzymes within the TCA).

3 TABLES

Supplementary Table 1. Basic characteristics of amplicon sequencing data.

	Replicate	Read pairs	Paired-end assembled reads[1]	Reads passing QC[2]	Number of OTUs[3]
RH1	1	1200503	1053248 (87,7%)	823870 (78,2%)	85220
	2	1421875	1181171 (83,1%)	919761 (77,9%)	114906
	3	1867327	1523755 (81,6%)	1163012 (76,3%)	79322
RH2	1	1716918	1477934 (86,1%)	1167223 (79,0%)	79364
	2	1146256	990484 (86,4%)	779733 (78,7%)	61351
	3	1764749	1530410 (86,7%)	1199066 (78,3%)	48352
RH3	1	1747498	1503555 (86,0%)	1188756 (79,1%)	72275
	2	1964018	1682628 (85,7%)	1331421 (79,1%)	77375
	3	1582135	1310726 (82,8%)	1095821 (83,6%)	72406
RHP	1	1102018	858439 (77,9%)	718994 (83,8%)	83526
	2	1152682	891388 (77,3%)	727199 (81,6%)	65581
	3	1239482	920613 (74,3%)	772622 (77,4%)	59547
Ref	1	1107732	998278 (90,0%)	820563 (82,2%)	171497
	2	1034858	893649 (86,4%)	734810 (82,3%)	121092
	3	1484880	1265663 (85,2%)	1044672 (82,5%)	127037

The assembly of read pairs and quality control were done using USEARCH (v. 7.0) (Edgar, 2010) as outlined above (1.4 Quality control and initial processing of amplicon sequencing data).

[1] Percentages refer to the proportion of read pairs that could be merged to paired-end reads.

[2] Percentages refer to the proportion of paired-end reads that passed QC.

[3] OTU numbers are based on OTU clustering applying an OTU threshold of 97% sequence identity. OTU numbers are based on datasets that have not been trimmed for singletons or doubletons.

Supplementary Table 2. Full-length 16S rRNA gene sequences reconstructed from metagenome datasets of sites RH1, RH2, and RHP.

Group	Sequence	Closest relative [NCBI Acc.]	Environment
DA052	DA052-RH1-1	AJ519366	U waste pile
	DA052-RH1-2	AY913232	acidic forest soil
	DA052-RH1-3	FJ466151	volcani deposit
	DA052-RH1-4	AJ519377	U waste pile
	DA052-RH1-5	AY963363	evergreen forest
	DA052-RH1-6	AY913381	acidic forest soil
	DA052-RH1-7	EF632750	Andean Altiplano, Lago Chungara
	DA052-RH2-1	KM235022	Tobacco rhizosphere
	DA052-RH2-2	AY913232	acidic forest soil
KF-JG30-18	KF-JG30-18-RH1-1	AY914560	acidic forest soil
	KF-JG30-18-RH2-1	AM749754	geothermal soil, NZ, ”Hell’s Gate”
DA111	DA111-RH1-1	AY913593	acidic forest soil
	DA111-RH1-2	AY913274	acidic forest soil
	DA111-RH1-3	DQ450765	alpine tundra wet meadow
	DA111-RH1-4	AY913230	acidic forest soil
	DA111-RH1-5	FJ800522	fresh water sponge
	DA111-RH2-1	DQ450765	alpine tundra wet meadow
	DA111-RH2-2	AJ295648	U waste pile
	DA111-RH2-3	AY963489	evergreen forest
	DA111-RH2-4	AY913593	acidic forest soil
	DA111-RH2-5	GU120600	asphalt lake
JG37-AG-4	JG37-AG-4-RH1-1	AY913277	acidic forest soil
	JG37-AG-4-RH1-2	FJ625337	lead contamined forest soil
	JG37-AG-4-RH1-3	EF018077	aspen rhizosphere
	JG37-AG-4-RH1-4	EF441915	Rio Tinto acid mine drainage
	JG37-AG-4-RH1-5	AY913277	acidic forest soil
	JG37-AG-4-RH1-6	EF018387	aspen rhizosphere
	JG37-AG-4-RH1-7	AY913277	acidic forest soil
	JG37-AG-4-RH2-1	FJ625337	lead contamined forest soil
	JG37-AG-4-RH2-2	EF018382	aspen rhizosphere
	JG37-AG-4-RH2-3	EF018387	aspen rhizosphere
	JG37-AG-4-RH2-4	EF441915	Rio Tinto acid mine drainage
	JG37-AG-4-RH2-5	EF516573	grassland soil
	JG37-AG-4-RH2-6	EU043812	inland dune, chronosequence
TM214	TM214-RH1-1	KF225682	acid mine drainage
	TM214-RH1-2	AF523913	coal rejects
	TM214-RH1-3	JQ311838	terrestrial microbiome
	TM214-RH2-1	JQ311838	terrestrial microbiome
	TM214-RH2-2	AB821189	lava-formed forest
	TM214-RH2-3	FJ625356	boreal pine forest

Continued on next page

Supplementary Table 2 – continued from previous page

Group	Sequence	Closest relative [NCBI Acc.]	Environment
	TM214-RH2-4	DQ450765	alpine tundra wet meadow
	TM214-RH2-5	AF523913	coal rejects
	TM214-RH2-6	HQ730692	Rio Tinto acid mine drainage
	TM214-RH2-7	DQ450765	alpine tundra wet meadow
	TM214-RH2-8	HQ420135	acidic coal mine drainage
	TM214-RH2-9	AB254776	Fe-oxyhydride nodules
	TM214-RHP-1	HQ420135	acidic coal mine drainage
	TM214-RHP-2	AB552456	volcanic ash deposits
	TM214-RHP-3	AB821091	lava-formed forest
	TM214-RHP-4	HE603995	iron-snow
	TM214-RHP-5	AB821189	lava-formed forest
	TM214-RHP-6	EU038011	acidic cave wall biofilm
	TM214-RHP-7	HQ420135	acidic coal mine drainage
	TM214-RHP-8	HQ420135	acidic coal mine drainage
	TM214-RHP-9	AF523913	coal rejects
	TM214-RHP-10	GU127806	acidic fen soil

16S rRNA gene sequences were reconstructed using EMIRGE (Miller et al., 2011). Closest related sequences were deduced by SINA searches (Pruesse et al., 2012) against the SILVA database (Quast et al., 2013) and phylogenetic analysis in ARB (Ludwig et al., 2004). Information about the environmental origin were taken from available publications or metadata deposited with the sequence information.

Supplementary Table 3. Overview of metagenome sequencing data.

	RH1	RH2	RHP
Read pairs	114534920	110684393	158713485
Reads passing QC	110068058 (96,1%)	105482227 (95,3%)	151730092 (95,6%)
Contigs >1kb	233821	192051	324763
Contigs >2 kb	79344	77288	126035
Contigs >5 kb	20393	24190	33750
Mapped reads	60766183 (55,2%)	79042967 (74,9%)	94694372 (62,4%)
	61008702 (55,4%)	79233987 (75,1%)	94763375 (62,5%)

Quality control and assembly was carried out as detailed in the main manuscript text using USEARCH (Edgar, 2010) and MEGAHIT (Li et al., 2014). Percentages refer to the proportion of read pairs that passed quality control (QC) and were mapped. mappings to assembled contigs were done using BBMAP (http://bio-bwa.sourceforge.net/). Values indicate mapped forward reads (first value) and mapped reverse reads (second value).

Supplementary Table 4. Basic characteristics of the metagenome-assembled genomes (MAGs) obtained from sites RH1, RH2, and RHP.

Name	Length [bp]	GC content [%]	Contigs	N50 [bp]	16S	Taxonomic affiliation	Completeness [%]	Contamination [%]
RH1 MAG 16a	35577612	63	48	116479	n	Subdivision 1	93,38	0,00
RH1 MAG 17	2680426	63	558	5671	n	Subdivision 1	75,86	2,75
RH1 MAG 18b	5057472	60	349	21174	y	Subdivision 13	90,67	50,86
RH1 MAG 19a	3698153	58	1543	2646	n	Subdivision 13	71,73	33,66
RH1 MAG 19b	3673822	57	288	18489	y	Subdivision 13	67,50	1,88
RH1 MAG 20	4885294	59	760	58735	y	Subdivision 2, DA052	93,97	5,15
RH2 MAG 14	6007197	61	585	18863	n	Subdivision 3	96,52	7,29
RH2 MAG 16a	4284605	61	587	8678	n	Subdivision 1	84,24	17,86
RH2 MAG 17b	2798462	58	169	25655	y	Subdivision 13, KF-JG30-18	89,39	2,14
RH2 MAG 17c	2378194	57	115	28172	n	Subdivision 13, KF-JG30-18	75,80	1,28
RH2 MAG 19b	2230467	58	457	6233	y	Subdivison 13	70,94	3,42
RH2 MAG 21	3867140	58	557	10245	y	Subdivision 13	86,84	6,48
RHP MAG 18	4829037	62	153	49508	y	Subdivision 3, Bryobacter	85,22	0,87

If possible, information about the taxonomic affiliation was deduced from both recovered 16S rRNA genes and a phylogenetic tree (maximum-likelihood tree with FASTTREE (v. 2.1.3)) (Price et al., 2010) based on a concatenated alignment of 31 single-copy marker genes identified with AMPHORA (v. 2.0) (Wu and Scott, 2012). Completeness and contamination of the MAGs were determined with CHECKM (v. 1.0.3) (Parks et al., 2015). MAGs labeled with a, b, or c resulted from a second binning step applied to resolve strain heterogeneity as outlined in the materials and methods. 16S = 16S rRNA gene, y = 16S rRNA gene recovered from putative genome, n = recovery of 16S rRNA gene from putative genome not possible.

REFERENCES

Benjamini, Y. and Hochberg, Y. (1995). Controlling the false discovery rate: a practical and powerful approach to multiple testing. *Journal of the Royal Statistical Society: Series B (Statistical Methodology)* 57, 289–300

Edgar, R. C. (2010). Search and clustering orders of magnitude faster than BLAST. *Bioinformatics* 26, 2460–1. doi:10.1093/bioinformatics/btq461

Edgar, R. C., Haas, B. J., Clemente, J. C., Quince, C., and Knight, R. (2011). UCHIME improves sensitivity and speed of chimera detection. *Bioinformatics* 27, 2194–2200. doi:10.1093/bioinformatics/btr381

Falk, F. (2002). *Ein vergessenes rheinisches Braunkohlenrevier: Braunkohlenbergbau unter Tage am Nordabfall des Siebengebirges* (Rheinlandia-Verlag)

Gregor, I., Dröge, J., Schirmer, M., Quince, C., and McHardy, A. C. (2016). PhyloPythiaS+ : a self-training method for the rapid reconstruction of low-ranking taxonomic bins from metagenomes. *PeerJ* 4, e1603. doi:10.7717/peerj.1603

Laczny, C. C., Sternal, T., Plugaru, V., Gawron, P., Atashpendar, A., Margossian, H. H., et al. (2015). VizBin - an application for reference-independent visualization and human-augmented binning of metagenomic data. *Microbiome* 3, 1. doi:10.1186/s40168-014-0066-1

Li, D., Liu, C. M., Luo, R., Sadakane, K., and Lam, T. W. (2014). MEGAHIT: An ultra-fast single-node solution for large and complex metagenomics assembly via succinct de Bruijn graph. *Bioinformatics* 31, 1674–1676. doi:10.1093/bioinformatics/btv033

Lin, J. (1991). Divergence Measures Based on the Shannon Entropy. *IEEE Transactions on Information Theory* 37, 145–151

Ludwig, W., Strunk, O., Westram, R., Richter, L., Meier, H., Yadhukumar, et al. (2004). ARB: a software environment for sequence data. *Nucleic Acids Research* 32, 1363–71. doi:10.1093/nar/gkh293

Macdonald, T. L. and Bruce Martin, R. (1988). Aluminum ion in biological systems. *Trends in Biochemical Sciences* 13, 15–19. doi:10.1016/0968-0004(88)90012-6

Martin, M. (2010). Cutadapt removes adapter sequences from high-throughput sequencing reads. *EMBnet.journal* 17, 10–12

Miller, C. S., Baker, B. J., Thomas, B. C., Singer, S. W., and Banfield, J. F. (2011). EMIRGE: reconstruction of full-length ribosomal genes from microbial community short read sequencing data. *Genome Biology* 12, R44. doi:10.1186/gb-2011-12-5-r44

Oksanen, J., Blanchet, F. G., Friendly, M., Kindt, R., Legendre, P., McGlinn, D., et al. (2016). *vegan: Community Ecology Package*. R package version 2.4-1

Parks, D. H., Imelfort, M., Skennerton, C. T., Hugenholtz, P., and Tyson, G. W. (2015). CheckM: assessing the quality of microbial genomes recovered from isolates, single cells, and metagenomes. *Genome Research* 25, 1043–55. doi:10.1101/gr.186072.114

Piña, R. G. and Cervantes, C. (1996). Microbial interactions with aluminium. *BioMetals* 9, 311–316. doi:10.1007/BF00817932

Price, M. N., Dehal, P. S., and Arkin, A. P. (2010). FastTree 2 – approximately maximum-likelihood trees for large alignments. *PLoS ONE* 5, e9490. doi:10.1371/journal.pone.0009490

Pruesse, E., Peplies, J., and Glöckner, F. O. (2012). SINA: accurate high-throughput multiple sequence alignment of ribosomal RNA genes. *Bioinformatics (Oxford, England)* 28, 1823–9. doi:10.1093/bioinformatics/bts252

Quast, C., Pruesse, E., Yilmaz, P., Gerken, J., Schweer, T., Yarza, P., et al. (2013). The SILVA ribosomal RNA gene database project: improved data processing and web-based tools. *Nucleic Acids Research* 41, D590–6. doi:10.1093/nar/gks1219

Schmieder, R. and Edwards, R. (2011). Quality control and preprocessing of metagenomic datasets. *Bioinformatics* 27, 863–4. doi:10.1093/bioinformatics/btr026

Schroeder, J. I. (1988). K+ transport properties of K+ channels in the plasma Membrane of *Vicia faba*s Guard Cells. *The Journal of General Physiology* 92, 667–683

Tukey, J. W. (1949). Comparing individual means in the analysis of variance. *Biometrics* 5, 99–114

Wood, D. E. and Salzberg, S. L. (2014). Kraken: ultrafast metagenomic sequence classification using exact alignments. *Genome Biology* 15, R46. doi:10.1186/gb-2014-15-3-r46

Wu, M. and Scott, A. J. (2012). Phylogenomic analysis of bacterial and archaeal sequences with AMPHORA2. *Bioinformatics* 28, 1033–1034. doi:10.1093/bioinformatics/bts079

Zambenedetti, P., Tisato, F., Corain, B., and Zatta, P. F. (1994). Reactivity of Al(III) with membrane phospholipids: a NMR approach. *BioMetals* 7, 244–252
